# Biokinetics of nanoparticles and susceptibility to particulate exposure in a murine model of cystic fibrosis

**DOI:** 10.1186/1743-8977-11-19

**Published:** 2014-04-24

**Authors:** Marianne Geiser, Tobias Stoeger, Marco Casaulta, Shanze Chen, Manuela Semmler-Behnke, Ines Bolle, Shinji Takenaka, Wolfgang G Kreyling, Holger Schulz

**Affiliations:** 1 Institute of Anatomy, Medical Faculty, University of Bern, CH-3012 Bern, Switzerland; 2 Institute of Lung Biology and Disease, Helmholtz Center Munich – German Research Center for Environmental Health, D-85764 Neuherberg/Munich, Germany; 3 Current address: Institute of Epidemiology II, Helmholtz Center Munich – German Research Center for Environmental Health, D-85764 Neuherberg/Munich, Germany; 4 Institute of Epidemiology I, Helmholtz Center Munich – German Research Center for Environmental Health, D-85764 Neuherberg/Munich, Germany

**Keywords:** Aerosol, Biokinetics, Cystic fibrosis, Energy filtering transmission electron microscopy, Inflammation, Inhalation, Iridium, Lung function, Nanoparticles, Titanium dioxide, Ultrafine particles

## Abstract

**Background:**

Persons with cystic fibrosis (CF) are at-risk for health effects from ambient air pollution but little is known about the interaction of nanoparticles (NP) with CF lungs. Here we study the distribution of inhaled NP in a murine CF model and aim to reveal mechanisms contributing to adverse effects of inhaled particles in susceptible populations.

**Methods:**

Chloride channel defective *Cftr*^TgH (neoim) Hgu^ mice were used to analyze lung function, lung distribution and whole body biokinetics of inhaled NP, and inflammatory responses after intratracheal administration of NP. Distribution of 20-nm titanium dioxide NP in lungs was assessed on ultrathin sections immediately and 24 h after a one-hour NP inhalation. NP biokinetics was deduced from total and regional lung deposition and from whole body translocation of inhaled 30-nm iridium NP within 24 h after aerosol inhalation. Inflammatory responses were assessed within 7 days after carbon NP instillation.

**Results:**

*Cftr* mutant females had moderately reduced lung compliance and slightly increased airway resistance compared to wild type mice. We found no genotype dependent differences in total, regional and head deposition or in secondary-organ translocation of inhaled iridium NP. Titanium dioxide inhalation resulted in higher NP uptake by alveolar epithelial cells in *Cftr* mutants. Instillation of carbon NP induced a comparable acute and transient inflammatory response in both genotypes. The twofold increase of bronchoalveolar lavage (BAL) neutrophils in *Cftr* mutant compared to wild type mice at day 3 but not at days 1 and 7, indicated an impaired capacity in inflammation resolution in *Cftr* mutants. Concomitant to the delayed decline of neutrophils, BAL granulocyte-colony stimulating factor was augmented in *Cftr* mutant mice. Anti-inflammatory 15-hydroxyeicosatetraenoic acid was generally significantly lower in BAL of *Cftr* mutant than in wild type mice.

**Conclusions:**

Despite lacking alterations in lung deposition and biokinetics of inhaled NP, and absence of significant differences in lung function, higher uptake of NP by alveolar epithelial cells and prolonged, acute inflammatory responses to NP exposure indicate a moderately increased susceptibility of lungs to adverse effects of inhaled NP in *Cftr* mutant mice and provides potential mechanisms for the increased susceptibility of CF patients to air pollution.

## Background

Air pollution continues to be a major threat to human health, accounting for an estimated 3.7 million premature deaths in 2012 [1]. The association between exposure to air pollution, especially to airborne particulate matter (PM), and a wide range of adverse health effects, particularly respiratory and cardiovascular effects, has been established by plentiful epidemiologic evidence, supported by mechanistic in vivo animal studies [2-4]. The susceptible populations are children, women in the reproductive age, the elderly and persons with pre-existing respiratory and cardiovascular diseases. While many studies have focused on the interaction of PM with asthma or chronic obstructive pulmonary disease (COPD) [5], relatively little is known about PM effects in patients with cystic fibrosis (CF).

Cystic fibrosis (CF) is caused by mutations in the gene encoding the CF transmembrane conductance regulator (CFTR) and results in altered ion transport regulation across epithelial membranes [6]. In lungs, defective CFTR leads to hyperviscous, adhesive airway mucus and ultimate failure of mucociliary clearance [7]. Recurrent infections with CF-specific bacteria with intense inflammatory responses contribute to irreversible lung injury [8]. The fine pulmonary structure of newborn CF infants is assumed to be normal. However, it has been suggested that the beginning of lung disease might precede the colonization with CF-related pathogens [9]. Furthermore, a significant heterogeneity in the severity of lung disease is observed in patients carrying the same mutation; the basis for the divergence between genotype and lung phenotype has been attributed to modifier genes, but also to environmental factors [10,11]. Evidence for a relationship between PM_10_ and PM_2.5_ pollution and pulmonary disease in CF was found in a cohort study with higher rates of pulmonary exacerbations in CF patients [12]. Potential mechanisms discussed are changes in the breathing pattern, which result in increased particle deposition in CF patients [13], or alterations of mucociliary and/or macrophage-mediated particle clearance [14,15].

Beside PM_10_ and PM_2.5_, ultrafine or nanoparticles (UFP, NP, particles with diameters < 100 nm) are of increasing concern [16]. Adverse health effects occur at current levels of exposure, and no threshold concentration has been identified below which air pollution poses no risk for human health [17,18]. In addition, nanotechnology industry generates daily new NP, which may become airborne at some stage of their life cycle and thus pose additional health risks.

There is evidence for decreased NP clearance from lungs of patients with chronic obstructive pulmonary disease (COPD) [19] and for impaired NP uptake by macrophages in COPD mice [20]. Hence, there are several factors which may contribute to an enhanced susceptibility of CF lungs to adverse effects of inhaled NP.

**Figure 1:**
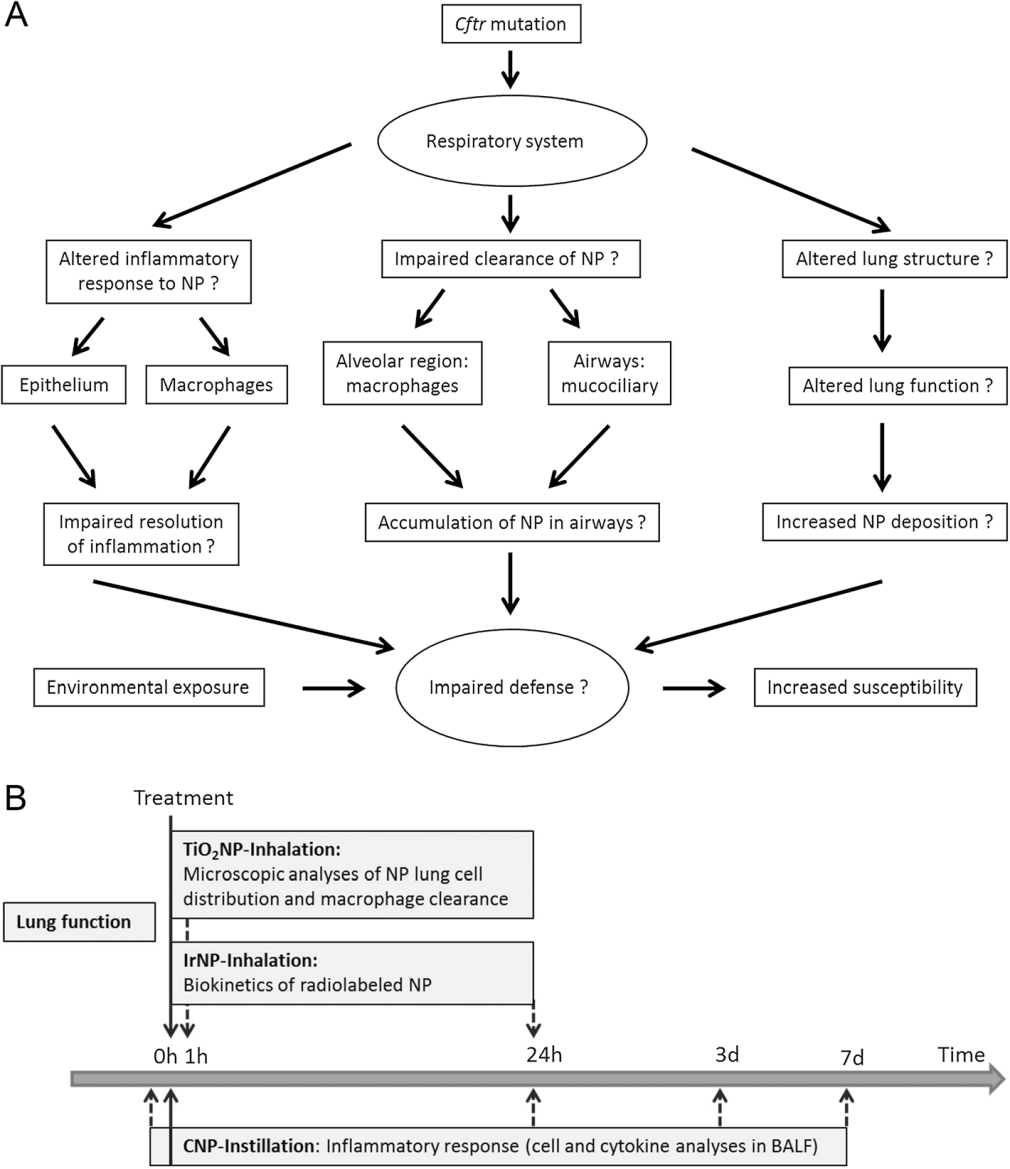
**Study motivation and experimental design. A)** Individual factors and multifactorial aspects for enhanced susceptibility of CF lungs to PM exposure. **B)** Time frame for lung function measurements, treatments with NP and subsequent analyses. CNP-Instillation: intratracheal instillation of carbon nanoparticles; BALF: Bronchoalveolar lavage fluid.

To evaluate individual factors and multifactorial aspects that may lead to enhanced susceptibility in CF lungs (Figure 1A), we investigated in a comprehensive approach (Figure 1B):

1. The distribution and clearance of inhaled 20-nm titanium dioxide NP (TiO_2_NP) at the individual particle level using analytical transmission electron microscopy (TEM) in chloride channel defective *Cftr*^TgH (neoim) Hgu^ mice in comparison to wild type (WT) littermates [21].

2. Inhaled radio-labeled iridium NP (IrNP) served to assess the biokinetics of NP.

3. We examined lung function, since its alteration may affect intrapulmonary particle deposition.

4. Lung exposure to carbon NP (CNP), an abundant component of urban, particulate air pollution served to evaluate the inflammatory responses as biological outcome.

## Results

### Lung physiology and fine pulmonary structures

Lung function data are summarized in Table 1. In general, body weight (BW) was lower in females than in males, but comparable among *Cftr* mutant and WT mice. In accordance with morphological studies [22,23], the mutation had no effect on lung size (TLC, total lung capacity), both, for absolute values and when expressed in relation to BW (TLC/BW). Similarly, the conducting airway volume (VD, series dead space volume) and its relative proportion to lung size (VD/TLC), as well as the gas exchanging capacity across the alveolar capillary membrane (D_CO_, diffusing capacity for carbon monoxide CO) were not altered, the latter also not when accounting for the alveolar volume (VA, D_CO_/VA). However, female *Cftr* mutants showed about 20% reduced respiratory system (C_RS_) and lung compliance (C_L_) values and an increased respiratory system resistance (R) compared to WT mice. These differences remained statistically significant when related to lungs size (C_RS_/TLC, C_L_/TLC, and specific respiratory system resistance, sR (R × TLC), respectively). Thus, the lungs of 3 months old female *Cftr* mutants are somewhat less distensible and put more resistance to airflow during breathing, which results in an increased work of breathing. Lung function of heterozygous females was not significantly affected, although there was a trend to limited compliance and resistance values. In male animals, differences between WT and *Cftr* mutant mice were noticeably for lung compliance (C_L_) and resistance (R), but they were of lower extent (10%) than in females and failed to be statistically significant.

**Table 1:** Lung function measurements in 14-weeks old mice

**Sex**	**Female**			**Male**		
**Genotype**	**WT**	**Cftr**^**+/−**^	**Cftr**^**−/−**^	**WT**	**Cftr**^**+/−**^	**Cftr**^**−/−**^
**N**	8	11	8	8	8	8
**BW (g)**	22.8 ± 1.8	21.7 ± 1.4	22.5 ± 1.9	32.0 ± 2.4	31.2 ± 1.8	29.5 ± 3.3
**TLC (mL)**	1.23 ± 0.12	1.21 ± 0.10	1.18 ± 0.09	1.28 ± 0.08	1.29 ± 0.06	1.30 ± 0.14
**TLC/BW (μL/g)**	54.4 ± 3.7	56.0 ± 3.6	52.8 ± 3.6	40.2 ± 2.9	41.4 ± 3.0	44.4 ± 6.4
**VD (μL)**	224 ± 15	230 ± 11	225 ± 11	231 ± 19	232 ± 13	241 ± 28
**VD/TLC (μL/mL)**	184 ± 9	191 ± 11	191 ± 8	181 ± 18	183 ± 6	186 ± 18
**C**_**RS **_**(μL/cm H**_**2**_**O)**	51.2 ± 7.1	51.8 ± 5.8	46.7 ± 6.7	52.9 ± 4.2	54.1 ± 4.3	54.0 ± 8.0
**C**_**RS**_**/TLC**	41.2 ± 1.9	42.7 ± 1.5	39.3 ± 2.8*	41.2 ± 1.4	37.1 ± 1.5	41.5 ± 1.8
**C**_**L **_**(μL/cm H**_**2**_**O)**	70.7 ± 16	64.3 ± 11.9	57.3 ± 7.0*	71.9 ± 10.3	72.2 ± 7.0	64.2 ± 9.4
**C**_**L**_**/TLC**	57.1 ± 12.5	52.8 ± 6.5	48.4 ± 4.6*	55.8 ± 5.4	56.5 ± 3.8	49.5 ± 5.8
**R (cm H**_**2**_**O/mL/s)**	0.78 ± 0.20	0.84 ± 0.12	1.00 ± 0.21*^+^	0.84 ± 0.14	0.76 ± 0.18	0.93 ± 0.15
**sR (cm H**_**2**_**O/s)**	0.84 ± 0.40	1.01 ± 0.11	1.16 ± 0.19*	1.08 ± 0.18	0.98 ± 0.23	1.19 ± 0.13
**D**_**CO **_**(μmol/min/hPa)**	11.2 ± 2.8	11.9 ± 2.0	11.5 ± 1.5	15.9 ± 2.2	15.4 ± 2.0	15.3 ± 1.6
**D**_**CO**_**/VA (μmol/min/hPa/mL VA)**	9.3 ± 1.7	9.8 ± 1.5	10.3 ± 0.8	12.0 ± 0.8	11.6 ± 1.3	11.6 ± 1.1
**D**_**CO**_**/VA (μmol/min/hPa/mL VA)**	9.3 ± 1.7	9.8 ± 1.5	10.3 ± 0.8	12 ± 0.8	11.6 ± 1.3	11.6 ± 1.1

Mean ± SD, WT: wild type, Cftr^+/−^: heterozygous *Cftr* mutants, Cftr^−/−^: homozygous *Cftr* mutants, BW: body weight, TLC: total lung capacity, VD: series (Fowler) dead space volume from helium expirogram, C_RS_: static compliance of respiratory system, C_L_: static lung compliance, R: respiratory system resistance, sR: specific respiratory system resistance (R × TLC), D_CO_: single breath diffusing capacity for carbon monoxide (CO), VA: alveolar volume determined from helium tracings of single breath diffusing capacity measurements, *: significant differences between aged matched wild type (WT) versus Cftr^+/−^ or WT versus Cftr^−/−^ , p < 0.05, ^+^: significant differences between age matched Cftr^+/−^ versus Cftr^−/−^, p < 0.05.

Also in agreement with the above mentioned morphological studies [22,23], we found no statistically significant differences in any of the lung structure parameters analyzed between *Cftr* mutant and WT mice. The lung tissue content in *Cftr* mutant mice was 15.0 ± 4.2% (range 12.4 – 19.8%). This pulmonary tissue consisted of 40.2 ± 6.7% connective tissue (range 33.3 – 46.8%), 33.4 ± 12.6% capillaries (range 19.1 – 43.3%) and 26.4 ± 9.2% epithelial cells (range 16.2 – 34.1%). In WT mice, 15.3 ± 2.3% was lung tissue (range 12.7 – 17.1%), which was composed of 27.8 ± 4.6% connective tissue (range 22.9 – 31.9%), 42.3 ± 6.6% capillaries (range 34.8 – 46.4%) and 29.9 ± 4.3% epithelial cells (range 25.0 – 33.3%).

### Cellular uptake of inhaled TiO_2_NP

A total of 469 - 659 hexagonal fields (equal to a volume of 6100 – 8600 μm^3^) on ultrathin lung sections were analyzed per animal for the presence of TiO_2_NP. Particles were first identified and morphologically localized by energy filtering transmission electron microscopy (EFTEM). NP uptake was further assessed by quantitative morphometry.

In *Cftr* mutants, we found a total of 8 TiO_2_ particles intracellularly, namely 5 NP in alveolar epithelial type 2 cells and 3 NP in macrophages, while in WT mice 7 particles were found, all in macrophages. Additional subsampling and analysis of 83 - 110 macrophages (on 2208 – 3260 hexagonal fields) per animal revealed 11 TiO_2_NP in *Cftr* mutants and 11 in WT mice. Hence, TiO_2_NP were found in alveolar type 2 epithelial cells as well as in macrophages in *Cftr* mutants and in macrophages only in WT mice (Table 2).

**Table 2:** **Sizes of TiO**_**2**_**NP and vesicles, distance of TiO**_**2**_**NP to vesicle membrane**

**Genotype**	**N**_**NP**_	**d**_**V**_**, nm**	**d**_**NP**_**, nm**	**R**_**V/P**_	**D**_**NP-M**_**, nm**
***Cftr *** **mutant**					
**Epithelium, type 2 cells**	5				
Mean ± SD		245 ± 92	37.7 ± 20.3	5.5 ± 1.4	47.6 ± 8.4
Range		168 - 347	22.6 – 66.2	4.2 – 7.0	36 - 56
**Macrophages**	14				
Mean ± SD		409 ± 327	36.7 ± 11.5	11.6 ± 10.8	28.2* ± 38.2
Range		101 - 1247	20.7 – 59.7	2.6 – 37.8	7 - 156
**WT**					
**Epithelium**	0				
**Macrophages**	18				
Mean ± SD		450 ± 256	40.7 ± 13.9	9.2 ± 4.6	58.1 ± 76.2
Range		183 - 857	27.4 – 86.0	2.8 – 17.1	11 - 267

N_NP_: Number of TiO_2_NP, d_V_: vesicle diameter, d_NP_: NP diameter, d_NP-M_: distance of NP to vesicle membrane, R_V/P_: ratio of vesicle to particle size, *: significant difference to WT mice, p < 0.05, n = 2 per genotype.

**Figure 2:**
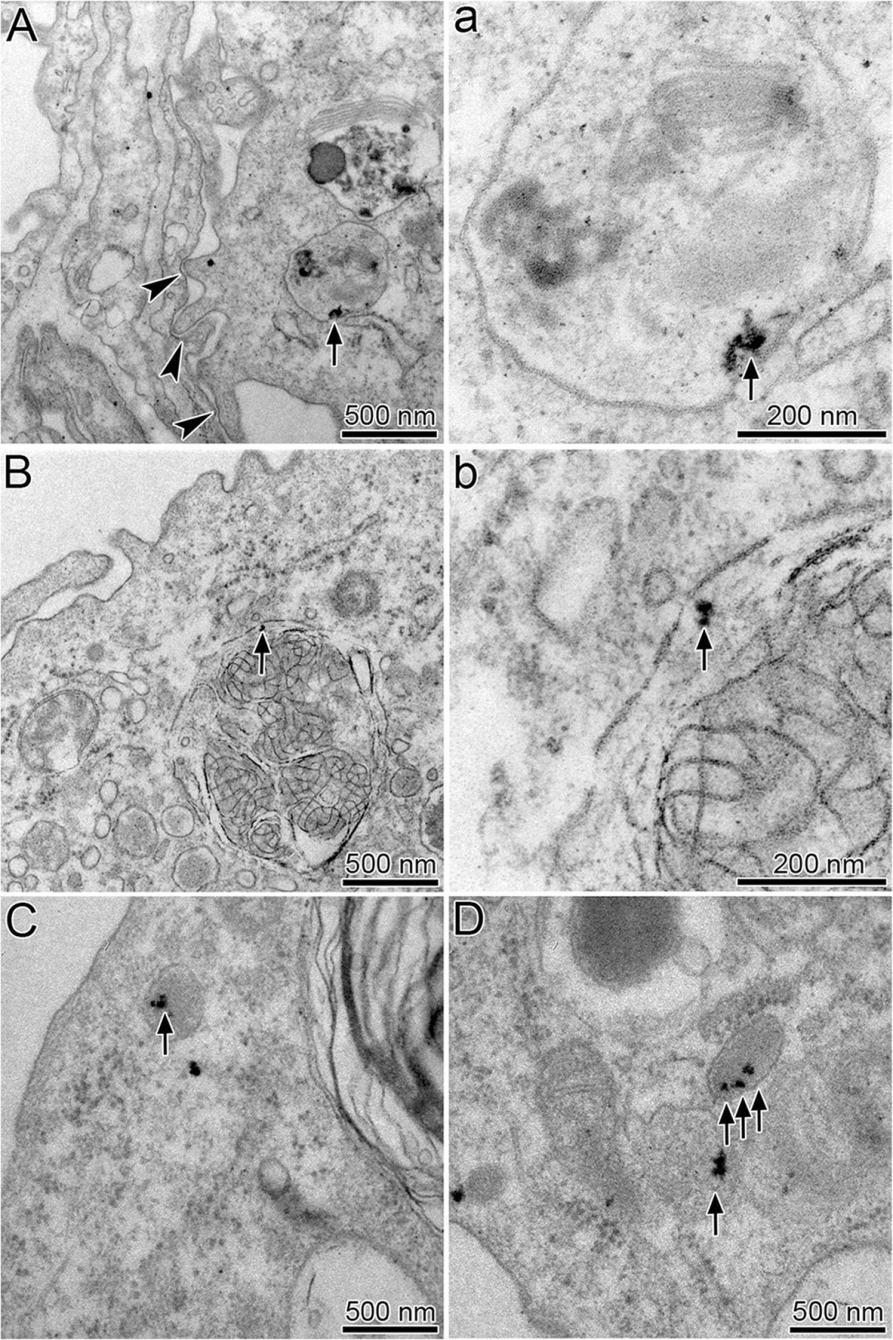
**Ultrastructural localization of TiO**_**2**_**NP in lung tissue.** TiO_2_NP (arrows): **A)** in a vesicle of a macrophage that is closely associated with the alveolar epithelium (arrow heads); **a)** high magnification micrograph of the NP-containing vesicle. **B)** in large vesicle of macrophage containing lamellar structures (surfactant); **b)** high magnification micrograph of the NP-containing phagolysosome. **C)** and **D)**, in vesicles of alveolar type 2 epithelial cells. Note the close association of the TiO_2_NP to the vesicle membrane.

In animals of both genotypes, TiO_2_NP were found in vesicles largely exceeding NP size (Figure 2, Table 2). Thus, the ratio of particle to vesicle size R_V/P_, which allows discrimination of endocytic uptake mechanisms [14,24], largely exceeded R_V/P_ ~ 1, i.e. the value representing phagocytic particle uptake. There were no differences observed in the diameters of NP-containing vesicles (d_v_) and internalized TiO_2_NP (d_NP_) between *Cftr* mutant and WT mice (Table 2). The distance of the particles to the vesicle membrane (d_P-M_), however, was with 28.2 ± 38.2 nm significantly (p = 0.026) smaller in macrophages of *Cftr* mutants compared to 58.1 ± 76.2 nm in WT mice. There were no significant differences in any of the parameters analyzed between NP in lung epithelial cells and in macrophages.

There was no particulate titanium found in control mice, which had been exposed to clean, filtered air.

### Biokinetics of IrNP

**Figure 3:**
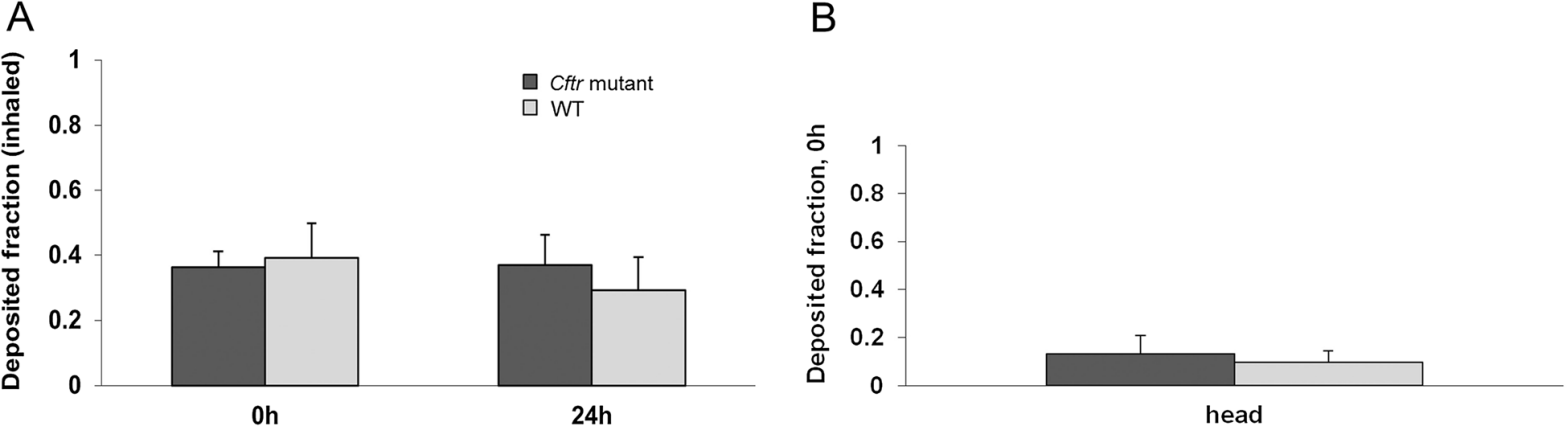
**Deposition of inhaled IrNP. A)** Total deposition determined either immediately (0 h) or 24 h after a 1-h inhalation exposure; given relative to the estimated inhaled aerosol. **B)** Head deposition determined immediately (0 h) after a 1-h inhalation exposure. No significant difference of total and head deposition between the two animal groups.

**Figure 4:**
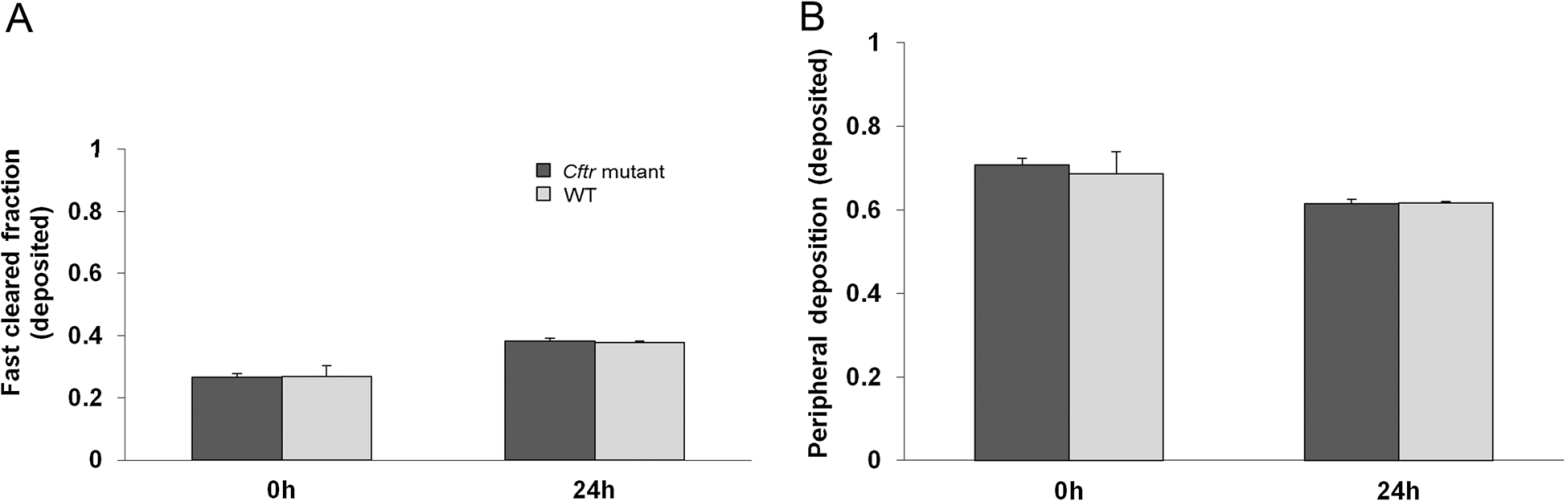
**Regional deposition of inhaled IrNP determined either immediately (0 h) or 24 h after a 1-h inhalation exposure.** The fast cleared fraction **(A)** estimates the airway deposition fraction including head airways. Regional deposition is given relative to the totally deposited aerosol. The difference between 0 h and 24 h relates to the fact that fast clearance is not finished after the 1-h inhalation. Therefore, only the 24-h values are to be considered. No significant difference between the two animal groups. Peripheral lung retention **(B)** represents long-term retained IrNP. The difference between 0 h and 24 h relates to the fact that fast clearance is not finished after the 1-h inhalation, adding to lung retention. Therefore, only the 24-h values are to be considered for IrNP retention in the peripheral lungs. No significant difference between the two animal groups.

**Figure 5:**
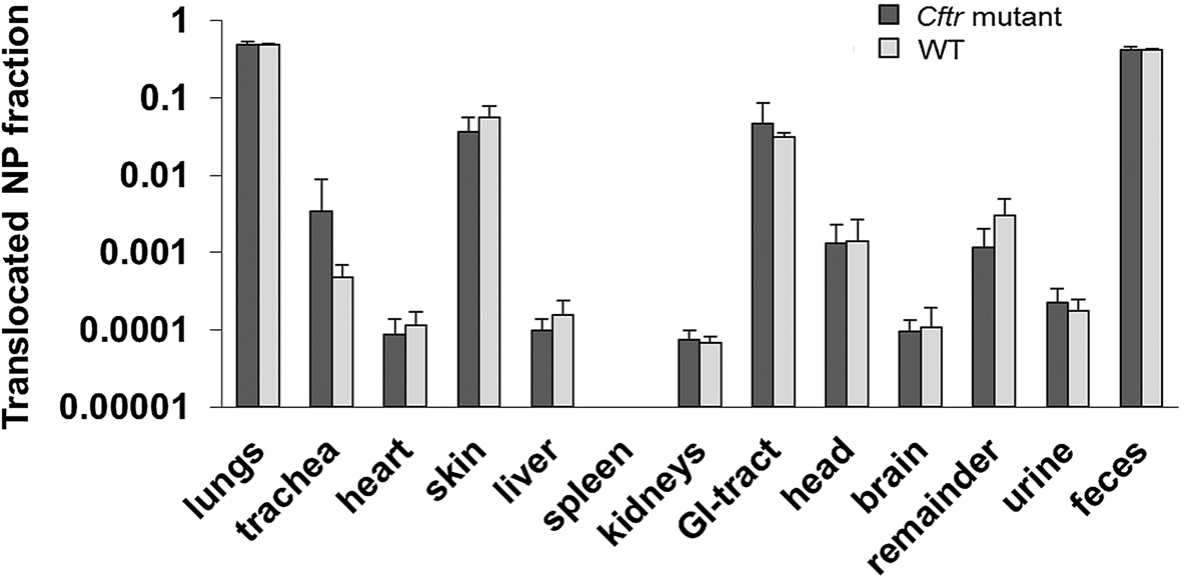
**Translocation of inhaled IrNP towards secondary target organs determined 24 h after the 1-h aerosol inhalation.** No significant difference between the two animal groups.

We found no significant differences in total, regional and head deposition of inhaled IrNP between *Cftr* mutant and WT mice (Figures 3 and 4). Inhaled NP translocation from lungs into systemic circulation and secondary target organs was also not different between the two genotypes, immediately (0 h) as well as 24 h after exposure to the NP aerosol (Figure 5).

### Inflammatory response to CNP

Histopathological examination of the lungs revealed focal accumulation of particle-loaded macrophages and granulocyte infiltrates with debris (alveolitis), which was most pronounced at day 1 after CNP instillation. There were no differences observed between *Cftr* mutant and WT mice.

**Figure 6:**
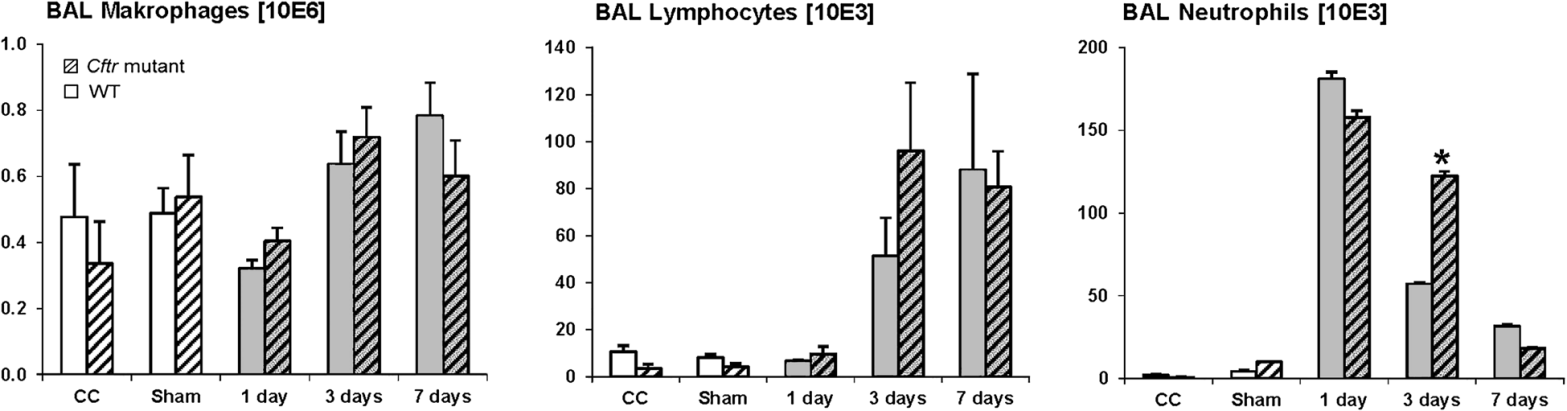
**BAL cells after CNP instillation.** Exposure to CNP led to a twofold increase of BAL neutrophils in *Cftr* mutants at day 3, but not at days 1 and 7. *: significant difference to WT mice, p < 0.05. CC: cage control (untreated animals); Sham: instillation of 50 μL pyrogene-free distilled water.

Total BAL cell numbers in untreated controls were comparable in both genotypes, i.e. 3.4 ± 0.6 × 10^5^ in *Cftr* mutants and 5.0 ± 1.6 × 10^5^ in WT mice. Intratracheal instillation of 20 μg CNP induced an acute BAL inflammatory cell infiltration dominated by neutrophils at day 1, which was equivalent in both animal groups (Figure 6). The subsequent resolution of the inflammation, analyzed at days 3 and 7 after CNP instillation, however, differed significantly between the two animal groups. BAL neutrophils decreased fivefold from 35% at day 1 to 7% at day 3 in WT mice, compared to a twofold decrease from 27% to 13% in *Cftr* mutants. Despite of this difference at day 3, neutrophils decreased to almost baseline levels, i.e. to 3% at day 7 in both animal groups. BAL lymphocyte and macrophage numbers increased after CNP instillation as well, though not statistically different between the two animal groups. In line with the lung response, peripheral blood monocytes were significantly increased at days 1 and 3 in *Cftr* mutant compared to WT mice, and levelled off at day 7 as well (data not shown).

**Figure 7:**
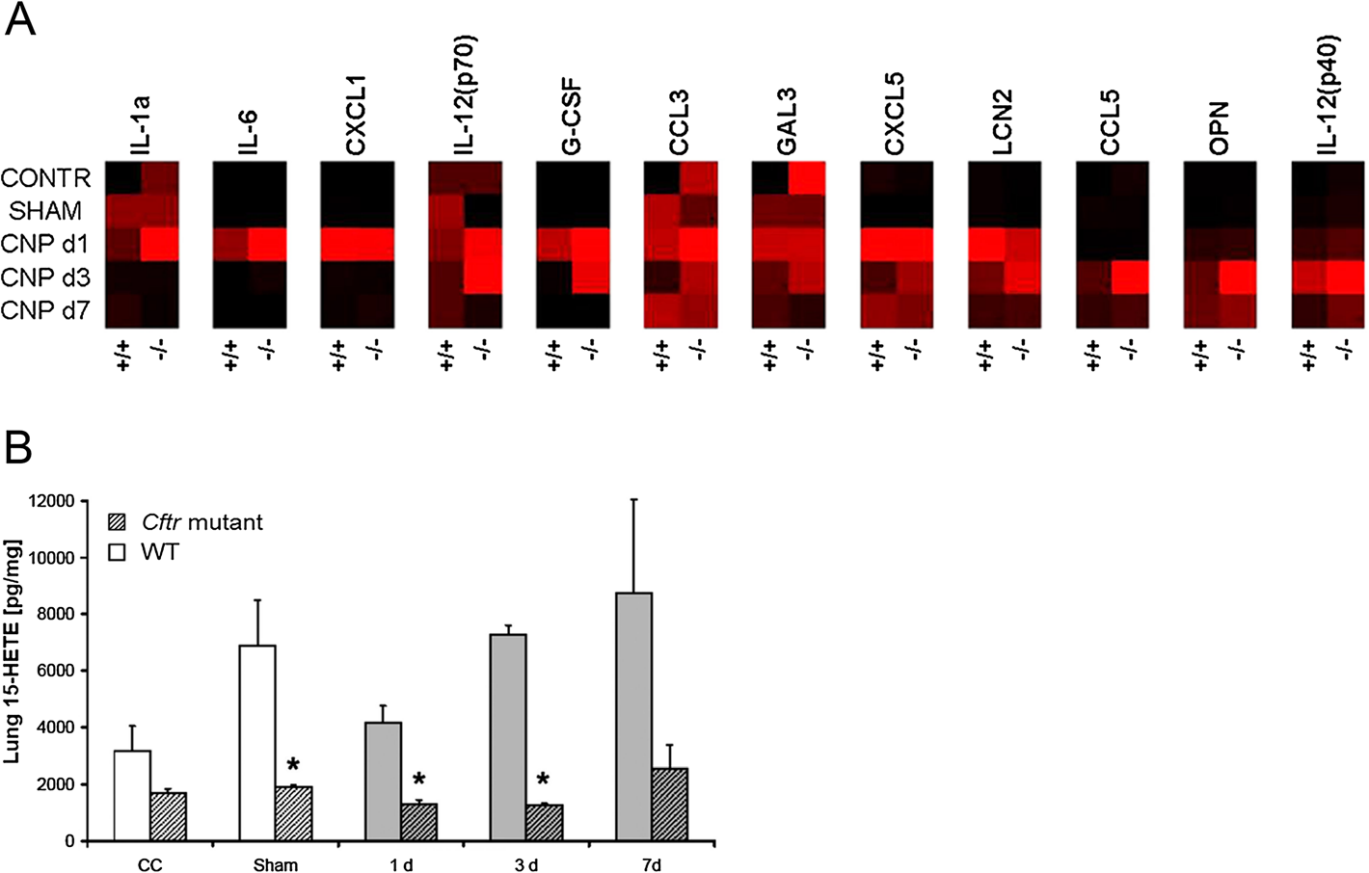
**BAL cytokines and lipid mediators after CNP instillation. A)** Heat map of cytokines. Black boxes indicate lowest and bright red highest detected levels for each cytokine (according to Table 3). Cytokines have been sorted by the response pattern. IL-1a, IL-6, and CXCL1 showed highest levels at day 1 after CNP exposure, CCL5, OPN, and IL-12p40 at day 3. Overall elevated cytokine levels were observed in *Cftr* mutant (−/−) as compared to WT (+/+) mice. **B)** 15-HETE. Increase over time after instillation treatment in WT mice, but independently of the CNP exposure; 15-HETE was generally reduced in *Cftr* mutant mice (hatched bars). *: significant difference to WT animals, p < 0.05. CC: cage control (untreated animals); Sham: instillation of 50 μL pyrogene-free distilled water.

The analysis of CNP-induced production of BAL cytokines (IL-1α, -6, -12p40, -12p70, G-CSF, CXCL-1, -5, CCL-3, -5) reflected the inflammatory response observed at the leukocyte level, whereby most cytokine levels were comparable between *Cftr* mutant and WT mice (Table 3, Figure 7A). At day 3 after CNP instillation, however, the inflammatory mediators G-CSF, CXCL5, CCL5, osteopontin (OPN/SPP1) and lipocalin-2 (LCN2) were significantly increased in *Cftr* mutant compared to WT mice (Table 3, Figure 7A/B). Most strikingly, G-CSF, which is critical for the survival of granulocytes, was 13 times higher in *Cftr* mutant compared to WT mice. This difference matches well with the prolonged high number of BAL neutrophils and indicates increased survival and poor clearance of BAL neutrophils in *Cftr* mutant mice. Additional immunohistological analysis indicated focal expression of osteopontin, a marker for inflammatory macrophages [25], at the site of accumulation of particle-loaded macrophages and accompanying cell debris.

**Table 3:** BAL cytokines

**A)**		**Cytokines (MultiPlex assay)**															
**Genotype**	**Treatment**	**IL-1a**		**IL-6**		**IL-12(p40)**		**IL-12(p70)**		**G-CSF**		**CXCL1**		**CCL3**		**CCL5**	
		pg/mL	*±*	pg/mL	*±*	pg/mL	*±*	pg/mL	*±*	pg/mL	*±*	pg/mL	*±*	pg/mL	*±*	pg/mL	*±*
**WT**	**CC**	3.5	*0.2*	5.7	*-*	1.4	*-*	4.0	*-*	1.2	*-*	2.5	*0.2*	49.3	*-*	1.6	*-*
	**Sham**	7.7	*2.4*	5.4	*0.3*	2.3	*0.3*	4.3	*0.2*	1.5	*0.3*	5.6	*2.5*	100.1	*28.6*	3.8	*1.3*
	**CNP d1**	6.4	*0.8*	54.4	*9.1*	4.5	*1.5*	4.2	*0.2*	105.5	*30.6*	104.4	*21.3*	103.7	*20.1*	2.4	*0.3*
	**CNP d3**	4.2	*0.5*	5.7	*-*	11.1	*8.1*	4.0	*-*	9.4	*3.6*	10.7	*4.0*	66.2	*12.7*	12.7	*6.4*
	**CNP d7**	4.9	*0.5*	5.7	*-*	4.2	*1.1*	4.0	*-*	1.3	*0.1*	8.6	*2.5*	100.9	*17.2*	9.0	*4.0*
***Cftr *** **mutant**	**CC**	6.9	*0.6*	5.7	*-*	2.6	*0.3*	4.0	*-*	1.2	*-*	3.6	*0.4*	101.4	*6.6*	5.1	*0.5*
	**Sham**	7.7	*0.8*	5.7	*-*	3.2	*0.3*	3.7	*0.3*	1.2	*-*	2.9	*0.3*	79.5	*15.0*	3.2	*0.7*
	**CNP d1**	11.7*	*3.7*	95.3	*29.8*	6.0	*1.3*	4.5	*0.2*	156.1	*59.0*	92.4	*24.0*	129.7	*22.2*	2.8	*0.4*
	**CNP d3**	4.2	*0.6*	14.0	*5.3*	15.4	*4.6*	4.7	*0.6*	125.3*	*73.5*	8.6	*1.0*	99.6	*18.3*	35.4*	*16.3*
	**CNP d7**	4.0	*0.5*	5.7	*-*	7.2	*1.8*	3.9	*0.2*	1.2	*-*	13.8	*1.7*	93.2	*28.9*	11.6	*3.8*
**B)**		**Cytokines (ELISA assay)**															
**Genotype**	**Treatment**	**CXCL5**		**LCN2**		**GAL3**		**OPN**									
		pg/mL	*±*	ng/mL	*±*	ng/mL	*±*	ng/mL	*±*								
**WT**	**CC**	70.0	*14.7*	20.9	*1.3*	7.0	*4.4*	5.9	*0.6*								
	**Sham**	22.5	*2.0*	22.4	*1.4*	14.1	*0.8*	4.7	*1.0*								
	**CNP d1**	423.7	*70.6*	226.0	*36.5*	18.8	*2.9*	12.3	*2.8*								
	**CNP d3**	145.7	*51.2*	101.8	*15.6*	13.1	*3.1*	20.3	*8.9*								
	**CNP d7**	241.1	*26.0*	63.9	*16.2*	12.2	*1.0*	21.2	*3.1*								
***Cftr *** **mutant**	**CC**	49.2	*4.2*	12.3	*1.4*	23.8*	*2.4*	5.8	*2.0*								
	**Sham**	25.1	*3.4*	8.7	*7.2*	13.9	*0.9*	6.3	*1.9*								
	**CNP d1**	413.5	*55.6*	163.7	*32.6*	19.0	*3.2*	13.8	*1.6*								
	**CNP d3**	252.5*	*31.5*	181.6*	*31.6*	17.9	*2.3*	46.2*	*4.8*								
	**CNP d7**	207.4	*50.1*	72.0	*11.1*	10.1	*3.1*	25.9	*2.8*								

Mean ± SD, *: significant difference to corresponding value in WT mice, p < 0.05, CC: cage control (untreated animals), Sham: instilled with 50 μL pyrogen-free distilled water.

BAL lipid mediator analysis revealed generally reduced levels of 15(S)-HETE, a major anti-inflammatory arachidonic acid metabolite from the 15-lipoxygenase pathway in *Cftr* mutants, statistically significant at days 1 and 3 after CNP instillation as well as after sham treatment (Figure 7B). The lipid mediators, LTB4, lipoxin-A4 and PGE2 remained unchanged (data not shown), except for 8-Isoprostan (8-IP), which was significantly reduced in *Cftr* mutants (48.6 ± 26.7 pg/mL) compared to WT mice (447.2 ± 219.5 pg/mL) at day 3.

## Discussion

In a comprehensive approach respecting possible multifactorial causes for enhanced susceptibility of CF lungs to environmental PM exposure (Figure 1), we evaluated whether structural or functional parameters of the *Cftr* mutant lungs were affected by the mutation. Overall, we have no indications for microstructural changes of the peripheral lung, which is in agreement with our previous findings [22,23]. While lung and airway volumes as well as gas exchange capacity were not affected by the mutation, we found moderate changes in lung resistance and compliance. Since structural changes were not detectable, an altered homeostasis of the pulmonary lining fluid may be a potential explanation. We also do not have indications for altered deposition or biokinetics of inhaled IrNP in the body.

However, despite finding an almost ‘normal lung’ in *Cftr* mutants, there is evidence for alterations in the cellular uptake of inhaled TiO_2_NP similar to findings we recently obtained for the uptake of inhaled 20-nm gold (Au) NP in Scnn1b transgenic mice (airway targeted overexpression of the epithelial Na + channel β subunit Scnn1b), which mimic key aspects of chronic obstructive lung diseases in humans [20]: (i) The uptake of a substantial part of the NP (26%) by alveolar epithelial cells in *Cftr* mutants points to less efficient TiO_2_NP uptake by surface macrophages in these mice. This may promote depth translocation of the NP, for which, however, we obtained no evidence from the data of the biokinetic study with IrNP. Enhanced interaction of NP with epithelial cells may induce or alter inflammatory responses, as we have observed -and which we discuss in detail below- in *Cftr* mutants after exposure to CNP. As in a previous inhalation study with 20-nm TiO_2_NP in rat lungs [26], we did not observe TiO_2_NP bound to the apical surface of epithelial cells. (ii) The localization of TiO_2_NP in vesicles largely exceeding NP size (R_V/P_ > > 1) in all cell types and in both animal groups indicates endocytic uptake of the NP. This finding is in agreement with other reports on intra-vesicular particle localization, suggesting NP uptake by cells to mainly occur by endocytic processes [27]. (iii) The close localization of NP to the vesicular membrane (d_NP-M_) indicates that deposited NP attached to the cell surface, resulting in internalization of the material by endocytosis. This correlates with findings in the above mentioned inhalation studies in rodents with TiO_2_ and Au nanoparticles [26,20]. In the latter study, fast attachment of AuNP to lung cells has been demonstrated [26]. Monocyte/macrophage expressed CFTR protein has recently been described to not only support phagolysosomal acidification but also contribute to the opsonization and phagocytosis of particles and bacteria [28]. Data showing the statistically significant reduction of d_NP-M_ in *Cftr* mutant compared to WT mice in the present study support this idea. The absence of CFTR protein in the macrophage membrane may have resulted in smaller particle-protein complexes, with possible consequences for the efficacy of particle uptake in macrophages. In addition, internalized particles would appear closer to the vesicle membrane. Besides receptor binding, adhesion forces may represent an alternative mechanism for the close localization of AuNP to the vesicle membrane [29]. In this case, however, we would expect d_NP-M_ values near 0. In addition, adhesion forces provide no explanation for the recorded difference in d_NP-M_ between *Cftr* mutant and WT mice.

**Figure 8:**
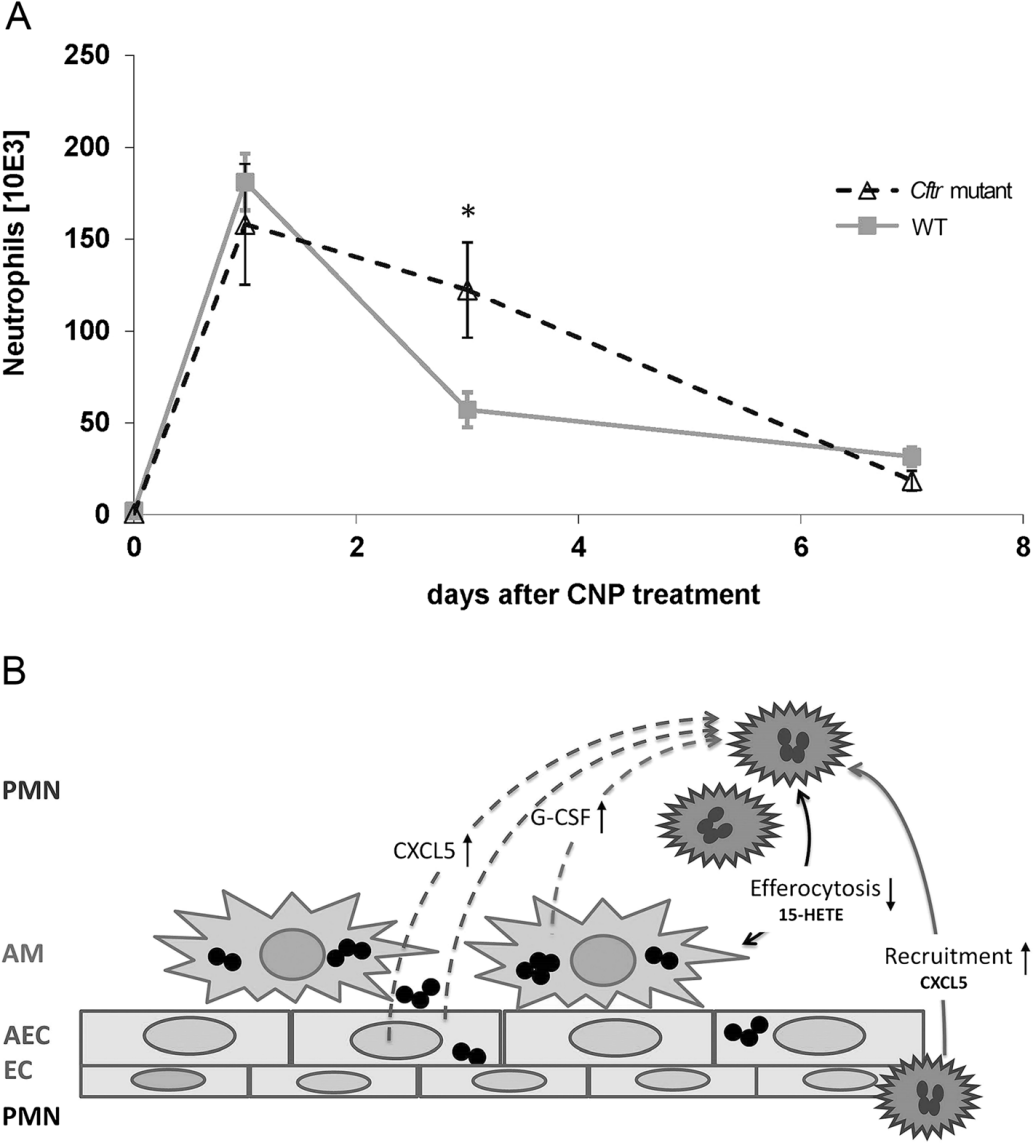
**Neutrophilic inflammation and its resolution in CF airways. A)** Time course of BAL neutrophils after CNP instillation showing a slower decline of BAL neutrophils from days 1 to 7 in *Cftr* mutant compared to WT mice. *: significant difference to WT animals, p < 0.05. **B)** Mode of action for impaired inflammation resolution in CF airways. The accumulation of inflammatory cells (PMN) in *Cftr* mutant lungs at day 3 after particle exposure is probably supported by different pathways: Augmented CXCL5 levels, released from *Cftr* mutant inflammatory epithelial cells, enhance exposure-related PMN recruitment and accumulation on the airway surface. In addition, elevated G-CSF and reduced 15-HETE levels in *Cftr* mutant lungs contribute to prolonged PMN survival and impaired efferocytosis of apoptotic PMN by macrophages. PMN: Polymorphnuclear cells = neutrophils, AM: Airway macrophages, AEC: airway and alveolar epithelial cells, EC: endothelial cells.

In addition to altered cellular uptake of TiO_2_NP in *Cftr* mutants, changes in the resolution of the inflammation in response to CNP exposure in these mice provide experimental support for the finding of an increased susceptibility of CF patients to environmental PM exposure [12]. While CNP instillation induced an acute alveolitis equally in *Cftr* mutant and WT mice at day 1, there were twice as much BAL neutrophils in *Cftr* mutant compared to WT mice at day 3. Thus, at day 3, 70% of the neutrophils were already removed in WT mice compared to 23% in *Cftr* mutants. The slower decline of BAL neutrophils indicates delayed or impaired resolution of the inflammation in *Cftr* mutant mice. This is further supported by the rate of the neutrophils’ decrease from day 3 to 7, which remained basically unchanged in *Cftr* mutants, whereas it was biphasic in WT mice (Figure 8A). Recruitment of macrophages and lymphocytes was apparently not affected in *Cftr* mutant mice, as the numbers of these cell types in BAL were similar in both animal groups.

Besides the acute response peaking at day 1, BAL cytokine profiling also indicated higher concentrations of G-CSF, CCL3, CCL5, CXCL5, LCN2, OPN and IL-12p40 at day 3 in *Cftr* mutant compared to WT mice. Thereby, the increases in CCL5, OPN and IL-12p40 in *Cftr* mutants indicate inflammatory-stimulated macrophages, which are important producers of G-CSF. Increased levels of CXCL5 and LCN2 indicate inflammatory stimulation of the epithelium attracting neutrophils. Consequently, increased G-CSF levels until day 3 supports the survival and persistence of the otherwise short-lived neutrophils in *Cftr* mutants. Thus, the congruent cellular and molecular data, which are summarized in Figure 8B, suggest that an impaired resolution of the inflammation causes increased neutrophil numbers at day 3 in *Cftr* mutant mice rather than escalating recruitment of inflammatory cells during the initiation phase. The generally significantly lower levels of BAL 15-HETE in *Cftr* mutant compared to WT mice further suggests an association of this lipid mediator with the compromised resolution of CNP-induced airway inflammation in *Cftr* mutants. The eicosanoid 15-HETE is not only expressed by leukocytes but also by the respiratory epithelium [30], and it is known as an anti-inflammatory and pro-resolving lipid mediator, which counteracts neutrophilic airway inflammation [31]. In humans, the 15-keto-PGE2–PPAR-γ system has been suggested to regulate abnormal mucus production in CF, with the unsaturated fatty acid derivate 15-keto-PGE2 and 15-HETE representing crucial PPAR-γ ligands [32].

In summary, despite lacking significant alterations in lung deposition and biokinetics of inhaled NP and absence of significant differences in lung function and structure, alterations in cellular uptake of inhaled TiO_2_ and the significantly enhanced, acute inflammatory responses to NP exposure indicate a moderately increased susceptibility of *Cftr* mutant lungs.

## Conclusions

Our results lead to the conclusion that the function of the CFTR gene, to the extend as investigated here in the *Cftr*^TgH (neoim) Hgu^ mouse line, is dispensable for lung function development as well as to mechanisms required for nanoparticle deposition or translocation. In contrast, the absence of CFTR appeared to modify cellular uptake of NP and caused a moderate impairment of the resolution of a by particle instillation induced neutrophilic airway inflammation. While the described effects might not be alarming for a single exposure to NP under aseptic conditions, the lungs of a CFTR deficient organism may be more susceptible to repetitive or chronic exposures to inflammatory stimuli like particulate air pollution and airway infection. Under these conditions, insufficiently resolved inflammation might promote the development of chronic inflammatory lung disease.

## Materials and methods

### Experimental design

To resolve mechanisms contributing to enhanced susceptibility to inhaled NP in *Cftr* mutants (Figure 1A), we studied (i) lung function prior to NP exposure, (ii) NP distribution in lung cells immediately (0 h) and 24 h after a 1-h exposure to 20-nm TiO_2_ aerosol, and (iii) NP biokinetics within 24 h after exposure to radio-labeled 30-nm Ir aerosol (Figure 1B). Finally, (iv) inflammatory responses were determined at days 1, 3 and 7 after intratracheal instillation of carbon NP (CNP, mean agglomerate size 210 nm), in *Cftr* mutant mice with no signs of lung disease and in WT littermates.

### Animals

#### Origin, genetics, husbandry

*Cftr*^TgH (neoim) Hgu^ transgenic (*Cftr*^tm1HGU^) mice created by targeted insertional mutagenesis have markedly decreased CFTR activity [21,33]. They survive to adulthood and have a phenotype resembling that of human compound heterozygotes. Although these mice – like most other CF mouse models – do not develop lung disease spontaneously, *Cftr*^tm1HGU^ mice develop pathogen-specific lung disease after bacterial challenge; they are more easily infected and develop more severe lung pathology than non-*cf* mice [34]. Uninfected *Cftr*^tm1HGU^ mice have a normal fine pulmonary structure including the lung lining layer [22,23].

*Cftr* mutant and WT mice (MF1, Charles River, Sulzfeld, Germany) were bread and reared in the animal facility of the Institute of Lung Biology and Disease at the Helmholtz-Center Munich, Neuherberg/Munich, Germany. Mice were housed in individually ventilated cages (IVC; BioZone, Ramsgate, UK) supplied with filtered air and access to food and water ad libitum. They were kept on a 12 h day/night cycle, humidity was maintained at 55% and the temperature of the control room was 22°C. Mouse progeny were genotyped by tail biopsy polymerase chain reaction (PCR) using the genotyping primers HGU-Neoplas-KO1: 5′-AAA GTG TAA AGC CTG GGG TGC-3′ and HGU-NeoAS-KO2: 5′-AGA ACT CGT CAA GAA GGC GAT AG-3′ generating a 3691 bp KO-product and HGU-Int9-WT1: 5′-GGA GCC TAG CAT AGA TGT CTC C-3′ and HGU-M10B-WT2: 5′-CTG CTG TAG TTG GCA AGC TTT GAC-3′ generating a 3274 bp WT-product (PCR conditions: 33× 94°C 30 sec, 64°C 30 sec, 72°C 2 min). Specified pathogen-free status was approved by a health certificate according to Federation of European Laboratory Animals Science Association (FELASA) guidelines.

Animals were treated humanely and with regard for alleviation of suffering. All experiments were conducted under federal guidelines for the use and care of laboratory animals and were approved by the Bavarian Animal Research Authority and by the Helmholtz Center’s Institutional Animal Care and Use Committee, as well as in accordance with the Swiss Federal Act on Animal Protection and the Swiss Animals Protection Ordinance.

### Nanoparticles

We selected the three different NP types for the following technical reasons: Biokinetics of inhaled NP particles: Iridium and titanium dioxide NP, since we previously developed reliable techniques of stably radio-labeling the IrNP [35,36] and elemental microanalysis by EFTEM for unambiguous identification of the TiO_2_NP [37]. The sensitivity of the radio-analysis allowed administering low doses in the μg range during a rather short 1-2-hour inhalation, thereby avoiding any toxicological side effects by the NP used. Besides the appropriate radio-analysis of both NP, identification of NP by EFTEM allowed elemental mapping of individual TiO_2_NP at the ultrastructural level in the tissue.

**Figure 9:**
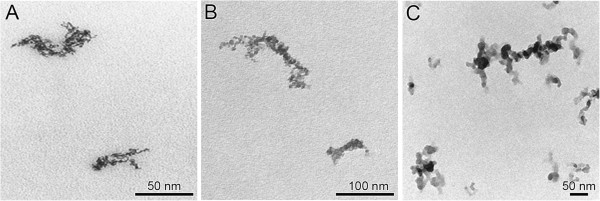
**Morphology of nanoparticles.** Chain-aggregated/agglomerated NP consisting of primary particles in the size range of about 5 nm (CNP: 5 - 12 nm). **A)** IrNP, **B)** TiO_2_NP and **C)** CNP.

Inflammatory response of the lung: The comparative investigation of toxicological effects of NP in *Cftr* mutant and WT mice required applying higher NP doses, such that an acute inflammatory response was induced. Since carbon nanoparticles are most relevant for environmental exposure, pure elemental CNP were administered by intratracheal instillation [38]. The latter method allowed reproducible and well controlled NP dosing, which was important to compare toxicological responses to NP in *Cftr* mutant and WT mice. However, this bolus application is less physiological than inhalation. The deposition pattern of instilled NP is different from that of inhaled NP. The target tissue of instilled and inhaled NP, however, remains the same, as NP are deposited on the epithelium of airways and alveoli. Since the lungs of our animals were “sterile” prior to NP treatments, there was no pre-existing lung pathology with excess mucus production. In addition, we have previously performed comprehensive characterization of all three NP (IrNP: [35,36]; TiO_2_NP: [37,39]; CNP: [38,40,41]). Thereby, we have shown that the morphology of all three particle types was very similar (see also Figure 9), namely chain-aggregated/agglomerated NP consisting of primary particles in the size range of about 5 nm (CNP: 5 - 12 nm).

### Lung distribution and clearance of inhaled TiO_2_ aerosols

Three *Cftr* mutant and three WT mice (female, aged 10 - 12 weeks, body weight 17.7 - 20.8 g) were used. There were no significant differences in age or body weight between mutant and WT mice. For particle inhalation, animals were deeply anaesthetized by intraperitoneal (i.p.) injection of a mixture of medetomidine (Domitor®, Pfizer GmbH, Karlsruhe, Germany; 500 μg/Kg), midazolam (Dormicum®, Hoffmann-La Roche AG, Grenzach-Wyhlen, Germany; 5 mg/Kg) and fentanyl (Fentanyl®, Janssen-Cilag GmbH, Neuss, Germany; 50 μg/Kg). For lung fixation immediately after inhalation, anesthesia was deepened with i.p. injection of a mixture of xylazin (Rompun®, Bayer Vital GmbH, Leverkusen; 5 mg/Kg) and ketamine (Ketamin 10%, WDT eG, Garbsen, Germany; 100 mg/Kg) prior to exsanguination by cutting the abdominal aorta. For 24-h examinations, anesthesia was antagonized by subcutaneous injection of atipamezole (Antisedan®, Pfizer GmbH Karlsruhe, Germany; 2.5 mg/kg), flumazenil (Anexate®, Hoffmann-La Roche AG, Grenzach-Wyhlen, Germany; 500 μg/Kg), and naloxone (Narcanti®, Janssen Animal Health, Neuss, Germany; 1.2 mg/Kg). For lung fixation at 24 h, a mixture of xylazin and ketamine was injected prior to euthanasia by exsanguination as above.

#### TiO_2_ aerosols, particle inhalation and deposition

TiO_2_ aerosol generation and inhalation were performed as previously described [39,26]. Briefly, ultrafine anatase TiO_2_ aerosols were generated with a Palas spark generator, quasi-neutralized with a radioactive ^85^Kr source, diluted and conditioned for inhalation in terms of gas composition, humidity and temperature. Particle size distribution and total number concentration were continuously monitored by a differential electrical mobility particle sizer (DMPS) and a condensation particle counter (CPC). The aerosol produced had a count median diameter (CMD) of about 20 nm (geometric standard deviation, GSD = 1.6) and a mean number concentration of 1 - 2 × 10^13^ particles/m^3^, resulting in a mass concentration of about 30 – 40 μg/m^3^. In addition, TiO_2_ aerosols were sampled for morphologic analysis on filters and on formvar-coated copper grids using a TEM particle sampler (Fachhochschule Nordwestschweiz FHNW, Windisch, Switzerland).

The deeply anesthetized mice were placed in airtight plethysmograph boxes for 1 h and inhaled the aerosols via an endotracheal tube by negative-pressure ventilation (-1.5 kPa) at a tidal volume of about 170 μl and a breathing rate of 120/min resulting in a minute ventilation of 20 cm^3^/min, which is 20% more than in spontaneously breathing mice. This inhalation protocol optimized NP deposition per exposure time. The deposited amount of TiO_2_ was calculated to be 0.1 μg or 7 × 10^9^ NP in each *Cftr* mutant or WT mouse lung (assuming a 40% deposition probability).

Two additional mice (one *Cftr* mutant and one WT) were not subjected to aerosol inhalation and served as controls.

#### Organ and tissue processing for microscopic analysis

To analyze the distribution of inhaled TiO_2_NP in lung cells at the individual particle level, organs were fixed in situ by instillation of phosphate-buffered 2.5% glutaraldehyde (350 mOsm, pH 7.4) at a pressure of 20 - 25 cm H_2_O as described previously [22]. Thereafter, lungs were removed *in toto* from the thorax and stored for at least 24 h in fixative solution at 4°C. Lung volumes [42] were 0.70 - 0.95 mL in *Cftr* mutant and 0.62 - 0.82 mL in WT mice and were not significantly different between the two animal groups.

Lungs were then subjected to systematic tissue sampling, post fixation with osmium tetroxide and uranyl acetate (Sigma-Aldrich, Buchs, Switzerland), dehydration in ethanol and embedding in epon (Sigma-Aldrich) as described previously [37,43]. Ultrathin sections (≤50 nm) were cut from 5 - 6 tissue blocks per animal, mounted onto formvar coated 600-mesh hexagonal copper grids and stained with uranyl acetate and lead citrate (Ultrastain; Leica, Glattbrugg, Switzerland).

TiO_2_NP were identified on ultrathin tissue sections by EFTEM using a LEO 912 TEM (Zeiss, Oberkochen, Germany) equipped with an in-column omega energy filter and operated at 120 kV as described previously [37]. Electron spectroscopic imaging (ESI) was performed with an energy slit width of 10 eV at the titanium L-edge at 464 eV using the three-window method implemented in the iTEM software (Olympus, Muenster, Germany). On each ultrathin section, we determined a random starting point from which we meander-like analyzed every fourth hexagonal field for the presence of TiO_2_NP and its anatomical-histological and (sub) cellular localization. In addition, on each ultrathin section all surface macrophages were sampled and analyzed for TiO_2_NP.

Finally, sections of ~ 1 μm thickness were cut from the sampled tissue blocks and stained with toluidine blue to evaluate the fine pulmonary structure by estimating the corresponding ratios with a point counting system.

### Lung physiology

Lung function was determined in anesthetized and intubated, 14-week old male and female *Cftr* mutant (n = 35) and WT (n = 16) mice as described previously [44,45]. It covers lung volumes, conducting airway volume, respiratory mechanics and gas exchange capacity. Body weight in females was significantly lower than in their male counterparts, the difference was 24% in *Cftr* mutants and 29% in WT mice (for details see Table 1).

### Deposition and biokinetics of inhaled IrNP

Sixteen female *Cftr* mutant and 16 WT mice (age: 10 - 12 weeks; body weight: 20 - 22 g) were used for biokinetic studies. Radiolabeled Ir (^192^Ir) aerosols of 30 nm CMD (GSD = 1.6) were generated as described for TiO_2_ above; the mean aerosol concentration was 3 × 10^6^ particles/cm^3^. Anesthetized mice inhaled the aerosol for 1 h during spontaneous breathing in a nose-only apparatus described previously for NP exposure of newborn rats [46]; about 2 × 10^9^ particles corresponding to 0.9 μg were deposited in the lungs. Mice were then treated as described above and euthanized either immediately (0 h) or 24 h after the 1-h aerosol inhalation.

Organs were harvested and Ir was analyzed quantitatively by gamma-spectrometry in all organs and tissue samples, as well as the remaining carcass and the excretion.

### Inflammation

Thirty nine *Cftr* mutant and 30 WT mice (female, age: 9 – 11 weeks, body weight: 19.1 - 22.3 g) were used to investigate the course of CNP induced lung inflammation.

#### CNP generation and instillation

CNP were generated by spark discharge from graphite electrodes (GFG1000, Palas, Karlsruhe, Germany) [47]. They mimic combustion-derived particles with low organic carbon content, large specific surface area (800 m^2^/g), as well as structural and compositional similarities to modern (Euro IV) diesel soot [48]. The physicochemical characteristics of CNP have been described in detail earlier [38,40,41]. Anesthetized mice were intubated by a nonsurgical technique and instilled with an aqueous suspension of 20 μg CNP in 50 μL pyrogen-free distilled water, followed by 100 μL of air [38,49]. The suspension of poorly soluble CNP was sonicated on ice for 1 min prior to instillation, using a SonoPlus HD70 (Bachofer, Berlin, Germany) at a moderate energy of 20 W to ensure sufficient particle dispersion with a mean agglomerate size of 210 nm (Zetasizer Nano ZS, Malvern Instruments, Herrenberg, Germany). Control animals were either instilled with 50 μL of pyrogen-free distilled water (Sham), or left undisturbed in their cages (cage control, CC).

#### Sample collection and preparation for inflammation analysis

Anesthetized mice were euthanized by exsanguination at days 1, 3 and 7 after CNP instillation. Blood was withdrawn from the retro orbital plexus with a capillary and collected in EDTA coated tubes (Sarstedt, Germany) for hematological analysis (ADVIA Hematology Systems (Bayer Diagnostics). Lungs were lavaged with 10 × 1 mL phosphate buffered saline (PBS, calcium and magnesium free) as described previously [38]. The BAL fluids from the first two lavages were used for protein analysis after cell separation by centrifugation (425 × g, 20 min at room temperature). The cell pellet was resuspended in 1 mL RPMI 1640 medium (BioChrome, Berlin, Germany) supplemented with 10% fetal calf serum (Seromed, Berlin, Germany). Cell viability was determined by the Trypan blue dye exclusion test. Differential cell counts were performed on cytospin preparations (May-Grünwald-Giemsa staining; 2 × 200 cells were counted).

#### BAL protein analysis

Total BAL protein content was determined spectrophotometrically with an ELISA reader (Labsystems iEMSReader MF, Helsinki, Finland) at 620 nm, using the Bio-Rad Protein Assay Dye Reagent (no. 500-0006; BioRad, Munich, Germany).

Cytokine analysis in BAL fluid was performed using the Bio-Plex bead-based suspension array system and the appropriate detection kits (Pro Mouse 10Plex, Bio-Rad, Hercules CA, USA) and specific ELISAs (mouse, R&D Duo Sets) for osteopontin (OPN/SPP1, catalog number DY441), galectin-3 (DY1197), LIX/CXCL5 (DY443) and lipocalin-2 (NCL2/NGAL, DY1857) as described previously [49].

Lung concentrations of the lipid mediators 15(S)-HETE and PGE2 were assessed as follows: Lung homogenates (about 50 mg lung tissue/500 μL) were prepared by disrupting lung tissue in HEPES buffer, pH 7.4, with lysing matrix E (FastPrep FP120 cell disrupter; MP Biomedicals Germany GmbH, Eschwege, Germany). After centrifugation (3200 × g at 4°C for 10 min), aliquots of the supernatants were taken for determination of protein and lipid mediators. Aliquots of supernatants derived from cell homogenates of particle-treated cells (prepared as described above) and those from lung homogenates were deproteinized by adding an 8-fold volume of 90% methanol containing 0.5 mM EDTA and 1 mM 4-hydroxy-2,2,6,6-tetramethylpiperidine-1-oxyl, pH 7.4 [50]. Methanol suspensions were stored at -40°C for 24 h followed by two centrifugation steps at 10,000 × g for 20 min at 4°C with a 24 h interval to remove the proteins. Aliquots of the obtained supernatants were dried in a vacuum centrifuge, dissolved in assay buffer and used for quantification of PGE2, Lipoxin-A4 and 15(S)-HETE by their specific enzyme immunoassays (Cayman Chemical Company, Ann Arbor, MI, USA) according to manufacturer’s protocol. Protein was measured at 595 nm using 5 μL of homogenate and 200 μL of 1:5 diluted BioRad solution (Bio-Rad, Munich, Germany) with bovine serum albumin as standard.

#### Histopathology and immunohistochemistry

Two to three mice of each group were sacrificed for histopathological and immunohistochemical examinations after CNP instillation. Lungs were fixed without lavage in phosphate buffered saline (pH 7.4) and 4% formalin at an inflation pressure of 20 cm H_2_O and embedded in paraffin.

Since we have previously shown that osteopontin (OPN/SPP1) expression is induced in alveolar macrophages at day 1 after CNP inhalation [25] and SPP1 concentration was found to be increased in BAL fluid, we performed immunohistochemical analysis of osteopontin in lung sections using a polyclonal antibody (R&D Systems, Wiesbaden, Germany). After staining with a biotinylated secondary antibody (Vector laboratories Inc., Burlingame, California, USA) and streptavidin-Vectastain Elite ABC peroxidase reagents (Vector Laboratories Inc.), lung sections were developed with Nova Red (Vector Laboratories Inc.) and counterstained with hematoxylin.

### Statistics

Lung physiology, deposition, distribution and biokinetics of inhaled NP: The non-parametric Mann–Whitney Test was used for two group comparisons and one-way analysis of variance (ANOWA) for multiple group comparisons. Inflammation: Two-way ANOVA was used to analyze differences between control and various exposure groups. Group means of WT and *Cftr* mutant mice were compared using the Student’s *t*-test. Overall, a significance level of p < 0.05 was adopted.

## Abbreviations

BAL: Bronchoalveolar lavage; BW: Body weight; C: Carbon; CF: Cystic fibrosis; CFTR: Cystic fibrosis transmembrane conductance regulator; CCL: Chemokine (C-C motif) ligand; CL: Lung compliance; CMD: Count median diameter; CNP: Carbon nanoparticles; COPD: Chronic obstructive pulmonary disease; CPC: Condensation particle counter; CRS: Respiratory system compliance; CXCL: Chemokine (C-X-C motif) ligand; DCO: Diffusing capacity for carbon monoxide; EFTEM: Energy filtering transmission electron microscopy; ESI: Electron spectroscopic imaging; G-CSF: Granulocyte-colony stimulating factor; HETE: Hydroxyeicosatetraenoic acid; i.p.: Intraperitoneal; IL: Interleukin; Ir: Iridium; IrNP: Iridium nanoparticles; LCN: Lipocalin; LDH: Lactate dehydrogenase; LTB: Leukotriene B; NP: Nanoparticle (s); OPN: Osteopontin; PCR: Polymerase chain reaction; PGE: Prostaglandin E; PM: Particulate matter; PPAR: peroxisome proliferator-activated receptor; R: Resistance; Scnn1b: Epithelial Na + channel β subunit; TiO2: Titanium dioxide; TiO2NP: Titanium dioxide nanoparticles; TLC: Total lung capacity; UFP: Ultrafine particles; DMPS: Differential electrical mobility particle sizer; SDg: Geometric standard deviation; VA: Alveolar volume; VD: Series dead space volume

## Competing interests

The authors declare that they have no competing interest.

## Authors’ contributions

The authors’ responsibilities were as follows – MG, TS, MSB, WGK and HS designed the research and were responsible for the experiments. All authors conducted specific parts of the research. MG, TS, WGK and HS wrote the manuscript. All authors critically read and approved the final manuscript.
